# Roles of Oxidative Phosphorylation and Fatty Acid Oxidation in Neuroinflammation Induced by Lipopolysaccharide in Hypothalamic Neuronal Cells

**DOI:** 10.1155/ijin/6298730

**Published:** 2026-04-15

**Authors:** Mohsine-Ali El-Hamri, Meriem Lahmouad, Jihane Zerrouk, Rafik El-Mernissi, Lhoussain Hajji, Khayelihle Brian Makhathini, Hanane Khalki, Oualid Abboussi

**Affiliations:** ^1^ Department of Biology, Mohammed V University in Rabat, Rabat, Morocco, um5a.ac.ma; ^2^ Faculty of Medicine and Pharmacy of Rabat, University Mohammed V, Rabat, Morocco, um5.ac.ma; ^3^ Department of Medical Genetics, National Institute of Hygiene, Rabat, Morocco, sante.gov.ma; ^4^ Department of Biology, Ibn Tofail University, Kenitra, Morocco, uit.ac.ma; ^5^ Department of Flow Cytometry Laboratory, National Institute of Hygiene, Rabat, Morocco, sante.gov.ma; ^6^ Bioactives and Environmental Health Laboratory, Moulay Ismail University, Meknes, Morocco, umi.ac.ma; ^7^ Department of Medical Biosciences, University of the Western Cape, Cape Town, South Africa, uwc.ac.za; ^8^ Department of Biology and Geology, Polydisciplinary Faculty of Beni Mellal, Sultan Moulay Slimane University, Beni Mellal, Morocco, universitesms.com

**Keywords:** gonadotropin-releasing hormone, neuroinflammation, neuronal cell metabolism, oxidative stress, synaptic plasticity

## Abstract

Neuroinflammation is intricately associated with impaired neuronal function and is a contributing factor in the development of neurodegenerative diseases. Significant alterations in cellular metabolism often accompany these inflammatory changes. Although considerable research has focused on understanding these metabolic shifts in astrocytes and microglia, the precise mechanisms linking neuroinflammation and cellular metabolism in neurons remain poorly understood. This study explores the connection between neuroinflammation and neuronal cell metabolism through a lipopolysaccharide (LPS)‐induced neuroinflammation model utilizing GT1‐7 hypothalamic neuron cultures. Our findings indicate that LPS‐induced neuroinflammation in GT1‐7 hypothalamic neurons is marked by reduced oxidative phosphorylation (OXPHOS) and decreased endogenous fatty acid oxidation (FAO). In contrast, exogenous FAO increases, leading to elevated ATP production, while glycolysis remains unchanged. These metabolic changes are associated with increased inflammatory markers (IL‐6, TNF‐α) and oxidative stress indicators (ROS, NO), as well as decreased synaptic plasticity (as indicated by synaptophysin) and impaired cellular function, as evidenced by reduced gonadotropin‐releasing hormone (GnRH) release. Our study highlights the intricate interplay between neuroinflammation and neuronal cell metabolism. These findings emphasize the significance of metabolic changes in neuroinflammatory processes, offering potential insights for therapeutic interventions in neurodegenerative diseases.

## 1. Introduction

Neurodegenerative diseases (NDDs) refer to a wide range of heterogeneous disorders with multifaceted origins [1]. Despite their distinct aetiologies, these conditions share standard features, such as age‐related neurodegeneration, oxidative stress, and mitochondrial dysfunction [2–4]. Moreover, NDDs are characterized by a persistent state of neuroinflammation and neurotoxicity, serving both as potential causes and consequences in the progression of these disorders [4, 5]. The concurrent presence of neurotoxicity and neuroinflammation, if sustained, can contribute to neuronal loss, ultimately leading to neurodegeneration [6, 7]. Neurons are particularly vulnerable in this scenario, and their demise is irreversible, defining neuronal death as the hallmark of neurodegeneration [7]. The specific NDD associated with these conditions is further determined by the affected central nervous system (CNS) region [1, 7, 8].

Lipopolysaccharide (LPS) is an important structural component of the outer membrane of Gram‐negative bacteria (GNB) and is highly neurotoxic [9]. LPS is associated with neurotoxicity and neuroinflammation in the CNS [10–12]. It activates microglia that release inflammatory molecules and generate reactive oxygen species (ROS) [12, 13]. This combined condition of neurotoxicity and neuroinflammation plays a pivotal role in the development and exacerbation of NDDs [9, 14].

Under inflammatory conditions, such as infections with GNB, LPS interacts with microglia’s Toll‐like receptor 4 (TLR‐4), activating them through the nuclear factor kappa B (NF‐kB) signaling pathways [12, 13]. Activated microglia release proinflammatory mediators such as tumor necrosis factor‐alpha (TNF‐α), interleukin‐1 (IL‐1), and IL‐6 [15]. These microglia‐derived cytokines stimulate quiescent astrocytes to become reactive [16]. Reactive astrocytes, in turn, release proinflammatory cytokines such as IL‐6, IL‐1, and IL‐15, along with chemokines such as C‐X‐C motif chemokine ligand 10 (CXCL10) [17–19], consequently, enhancing the inflammatory response by recruiting peripheral immune cells [20, 21]. However, these inflammatory responses are associated with metabolic changes observed mainly in astrocytes and microglial cells [16, 22, 23].

While TLR4 is traditionally recognized for its expression in microglia, recent studies have confirmed its presence in other CNS cells, including neurons [24, 25]. These findings challenge previous assumptions about neuronal TLR4 expression under normal conditions [24, 26]. It is important to note that the cellular response to LPS, including the induction of the Warburg effect and the adoption of pro or anti‐inflammatory phenotypes, is highly time‐dependent [27–30]. In our study, GT1‐7 hypothalamic neurons were exposed to LPS for 12 h. This time frame was chosen based on preliminary experiments and literature indicating that significant inflammatory and metabolic changes occur within this period, allowing us to capture the acute effects of LPS on neuronal metabolism and function [27, 31–33]. Activation of TLR4 in neurons can trigger proapoptotic signaling pathways, rendering them vulnerable to injury [34, 35]. Specifically, neuronal TLR4 expression may predispose neurons to apoptosis in the presence of stimuli such as the amyloid beta (Aβ) peptide or 4‐hydroxynonenal [36]. Moreover, TLR4 signaling in neurons can modulate AMPA receptor currents, influencing neuronal excitability [37]. Recent research has also found high TLR4 expression in cerebellar Purkinje neurons, demonstrating its involvement in maintaining motor coordination through nonimmune pathways [38]. Furthermore, TLR4 activation can promote neuronal differentiation and survival [39].

Neurons are less studied under inflammatory conditions, particularly regarding their metabolic profiling. However, they are known to follow a distinct metabolic pathway [40–42]. Under basal conditions, neurons primarily consume lactate to produce energy, a pathway highlighted by Pellerin and Magistretti (1994) [40, 43]. This model suggests that the high energy demands of neurons are met by lactate supplied from astrocytes. A longstanding debate exists over whether neurons prefer glucose or lactate as their substrate, with recent evidence supporting lactate utilization in the oxidative phosphorylation (OXPHOS) pathway [41, 42, 44, 45]. Despite this preference, other pathways, including glycolysis, β‐oxidation, and amino acids such as glutamine in gluconeogenesis, also contribute to neuronal metabolism [46–50]. While the OXPHOS pathway enhances energy production, it can increase toxicity due to elevated free radicals (reactive oxygen/nitrogen species: ROS/RNS), leading to oxidative stress [51, 52]. This imbalance between high oxidation rates and low antioxidant capacity can result in neuronal loss and contribute to neurodegeneration [53].

The literature suggests that neurons are “dependents” as they rely on astrocytes for energy and microglia for protection [46, 47, 54–57]. However, these conclusions are drawn from triple co‐cultures of neurons, microglia, and astrocytes or co‐cultures of neurons with either astrocytes or microglia.

Nonetheless, the exploration of neurons in monoculture to validate these assumptions has been lacking. Additionally, under stressful conditions such as inflammation and toxicity, neurons may adopt novel strategies to sustain their energy supply and survive independently of glial cells.

Furthermore, when lactate is scarce, neurons may be able to utilize alternative substrates and energetic pathways. Neuronal cells might also develop antioxidant strategies to navigate stress conditions.

Thus, we aim to investigate how the GT1‐7 hypothalamic neurons may adapt metabolically to LPS‐induced neuroinflammation in a monoculture setting. Our focus is on understanding the effects of LPS on neuronal metabolic preferences, identifying the pathways involved under inflammatory stress, and exploring the resulting impacts on synaptic plasticity and endocrine functionality.

## 2. Methods

### 2.1. GT1‐7 Mouse Hypothalamic Gonadotropin‐Releasing Hormone (GnRH) Neuronal Cell Culture

The GT1‐7 is an immortalized mouse hypothalamic cell line that is characterized by an increased GnRH release (SCC116, Sigma‐Aldrich, MA) [58]. The cells were made in culture using Dulbecco’s modified Eagle’s medium (DMEM; Gibco). For optimal cell culture, the following ingredients were added to the media: 10% fetal bovine serum (FBS; ThermoFisher), 45 mM glucose, 1 mM palmitate, and 100 μg/mL penicillin/streptomycin (P/S). The cells were incubated in a standard tissue culture incubator under humidified conditions (37°C, 5% CO2). Cells were maintained in complete DMEM (glucose concentration, 25 mM).

Our cell culture experiments were carefully designed with rigorous controls, including experimenters blinded to treatment conditions and automated data collection, to ensure unbiased, standardized procedures throughout the experimental process.

### 2.2. LPS Preparation and Treatment

LPS from Salmonella Minnesota (catalog number 437628, Calbiochem, Sigma‐Aldrich) was used as a Toll‐like receptor 4 (TLR4) signaling stimulator to stimulate the GT1‐7 cells. A stock solution of LPS was prepared at 5 mg/mL in sterile phosphate‐buffered saline (PBS). The solution was then filtered through a 0.22 μm filter to ensure sterility. Initial dilutions were made to create a range of LPS concentrations (0.05, 0.1, 0.2, 0.5, and 1 μM) for dose–response testing using the MTT assay, which assesses the reduction capacity of the cells (described in the subsequent section). Based on the MTT assay results, a final LPS concentration of 0.2 μM was selected, representing the highest dose that did not significantly reduce the MTT reduction capacity in GT1‐7 cells, suggesting minimal impact on cell viability. For subsequent experiments, GT1‐7 cells were treated with 0.2 μM LPS in serum‐free media for 12 h. Control cells were treated with serum‐free media alone.

### 2.3. MTT Reduction Assay

The viability of GT1‐7 cells was evaluated using the MTT assay (3‐(4,5‐dimethyl‐2‐thiazolyl)‐2,5‐diphenyl‐2H‐tetrazolium bromide) [59]. This assay measures the reduction of MTT to formazan by cellular reductases, serving as an indicator of mitochondrial activity. A decrease in MTT reduction suggests a decline in metabolic activity, which can be associated with reduced cell viability but does not directly measure cell survival. Absorbance at 490 nm was used to assess MTT reduction, with higher absorbance indicating more significant metabolic activity. This method allowed quantitatively comparing cellular metabolic activity between treated and control groups [59, 60].

Cells were seeded in 96‐well plates at a density of 4 × 10^4^ cells per well and exposed to either warm media (maintained at 37°C) or LPS at a concentration of 0.2 μM for 12 h. After treatment, the cultures were incubated at 37°C for 4 h in media containing 0.5 mg/mL MTT. Following incubation, cells were washed twice with PBS, and formazan crystals were solubilized using 100 μL of dimethyl sulfoxide (DMSO) per well. The plate was gently shaken for 10 min to ensure complete dissolution of the crystals. Absorbance was then measured at 490 nm using the Molecular Devices FlexStation II microplate reader.

### 2.4. Metabolic Profiling of GT1‐7 Cells: Assessing Bioenergetic Parameters and Mitochondrial Function

For a comprehensive bioenergetic and metabolic profiling of GT1‐7 cells, we used the Seahorse Metabolic Bioanalyzer XFe24 (Agilent; USA) [61]. This instrument measures the main important metabolic parameters. This included oxygen consumption rate (OCR) as an indicator of OXPHOS and fatty acid oxidation (FAO), as well as the extracellular acidification rate (ECAR) to evaluate glycolysis. These assessments were conducted following the manufacturer’s instructions (Agilent Technologies, 2016, 5991‐7138EN, USA) [61, 62].

GT1‐7 cells were seeded in pre‐PLL‐coated 24‐well Seahorse XF24 plates at a density of 4 × 10^4^ cells per well in a final volume of 400 μL GT1‐7 plating media, 24 h before any treatments. The wells were supplemented with DMEM (containing 10% v/v FBS, 200 U/mL penicillin–streptomycin, 8 mM L‐glutamine, and 7.5 mM glucose). More than 12 h before and after treatment with LPS or vehicle, the cells were washed with Seahorse XF DMEM medium (pH 7.4; Agilent) and supplemented with specific components such as L‐glutamine (2 mM), glucose (2.5 mM), and sodium pyruvate (2.5 mM) for the Mito Stress test. For the Glycolytic Stress test, the medium contained L‐glutamine at 2 mM. In the FAO assay, the cells were washed and then seeded in FAO assay medium containing 111 mM NaCl, 4.7 mM KCl, 1.25 mM CaCl2, 2 mM MgSO4, 1.2 mM NaH2PO4, supplemented with 2.5 mM glucose, 0.5 mM carnitine, and 5 mM HEPES at a final pH of 7.4. Following a 1‐h incubation in a non‐CO2 incubator at 37°C, we assessed glycolytic rate, oxidative metabolism, and FAO. These parameters were determined by monitoring ECAR and OCR, utilizing the Glycolytic Stress test kit, Mito Stress kit, and the XF Palmitate–BSA FAO Substrate kit (Seahorse Bioscience), as recommended by the manufacturer [63].

For the Glycolytic Stress test, ECAR measurements were obtained by exposing GT1‐7 cells to Seahorse XF DMEM medium containing 2 mM L‐glutamine (pH 7.4; Agilent), supplemented with 10 mM glucose as an energetic substrate. Second, we introduced oligomycin (1 μM), an ATP synthase inhibitor, to stimulate anaerobic glycolysis, followed by 2‐deoxyglucose (2‐DG; 50 mM) to inhibit glycolysis. Glycolytic rate, glycolytic capacity, and glycolytic reserve were calculated from the ECAR measurements [61, 64].

For the Mito Stress test, mitochondrial function was assessed by measuring OCR in cells incubated in Seahorse XF DMEM medium (2 mM L‐glutamine; pH 7.4; Agilent). A series of drugs, including (1) oligomycin (0.5 μM), which blocks the proton channel of ATP synthase, necessary for transforming ADP to ATP by OXPHOS, accomplishing the inhibition of ATP synthase; (2) carbonyl cyanide‐4‐(trifluoromethoxy) phenylhydrazone; (3) OXPHOS uncoupler (FCCP; 1 μM), a potent uncoupler of OXPHOS in mitochondria that disrupts ATP synthesis by transporting protons across cell membranes, used to detect maximal respiration in GT1‐7 cells; and (4) rotenone (R) with antimycin A (A), a respiratory complex III inhibitor (R/A; 0.5 μM), were added to the medium. Various mitochondrial respiratory parameters were calculated following the manufacturer’s guidelines, including basal respiration (BR), ATP production, proton leak, maximal respiration, spare capacity, and coupling efficiency (CE) [62, 63].

To evaluate FAO, OCR measurements were obtained in cells incubated in FAO media. Control wells were pretreated with a CPT1 irreversible inhibitor of FAO, etomoxir (40 μM), 15 min before the assay. Finally, BSA, used to assess utilization of endogenous fatty acid, or palmitate–BSA, used to assess utilization of exogenous fatty acid, was introduced to the appropriate wells immediately before the assay was conducted. The same parameters assessed in the Mito Stress test were calculated for the FAO test after the addition of oligomycin (2.5 μg/mL), FCCP (2 μM), rotenone (2 μM), and antimycin A (2 μM) [65, 66].

### 2.5. Quantification of Nitric Oxide (NO) Level

euronal NO production was evaluated by measuring accumulated nitrite released in the media. A cell suspension was prepared by seeding the GT7 cells in a 6‐well culture plate at a density of 1 × 106 cells/well (2 mL medium/well). After 24 h of pretreatment with saline or LPS, the supernatant was collected, and NO production was determined using an NO assay kit protocol. The absorbance of the reaction mixtures was measured at 540 nm using a FlexStation II microplate reader [67].

### 2.6. Quantification of Cellular ROS

The total intracellular superoxide and hydroxyl radicals were detected using the Cellular Reactive Oxygen Species Detection Assay Kit (No. ab186027, Abcam, US). The control and experimental group cells were seeded into a 96‐well plate at 2 × 10^4^ cells per well and incubated for 60 min in redwork solution at 37°C. The fluorescence signal was examined at Ex/Em = 520/605 nm using a Flexstation II microplate reader [68].

### 2.7. Measuring Cytokine Concentration and GnRH Release by Enzyme‐Linked Immunosorbent Assay

To measure cytokine concentrations, culture supernatants of GT1‐7 cells were collected after 12 h of LPS (0.2 μM) treatment in serum‐free media. Following treatment, the culture media were carefully aspirated and centrifuged at 3000 rpm for 10 min at 4°C to remove cell debris. The clarified supernatants were then aliquoted and stored at −80°C until further analysis. The concentration levels of TNFα, IL‐6, and GnRH were determined using ELISA kits (ab100747, ab222503, and DEIA‐BJ2472, respectively) according to the manufacturer’s instructions to ensure accurate and reproducible quantification.

### 2.8. Western Blot Analysis of Synaptophysin Expression

Western blot analysis of synaptophysin expression is more detailed in [69]. Briefly, GT1–7 cell proteins were extracted using RIPA buffer supplemented with protease inhibitors. After centrifugation at 3500 x g for 10 min at 4°C, the resulting supernatant containing the proteins was collected, and their concentrations were determined using the Bradford method [70, 71]. Subsequently, samples were denatured at 95°C for 5 min. Equal amounts of protein (20 μg) were then separated by electrophoresis on a 10% SDS polyacrylamide gel using BioRad running buffer at 200 V for 60 min. Transfer of proteins to a nitrocellulose membrane was performed using a Bio‐Rad transfer buffer at 100 V for 60 min. The membrane was blocked for 2 h with Li‐COR blocking buffer, followed by overnight incubation at 4°C with specific primary antibodies against synaptophysin (Cell Signaling, USA; catalog no. #5461) and β‐actin (Cell Signaling, USA; catalog no. #3700) at a 1:1000 dilution in blocking buffer. After washing thrice with 0.1 M PBS–Tween for 10 min each, the membrane was incubated with secondary antibodies (IRDye 800CW goat antirabbit and IRDye 680RD goat antimouse, LI‐COR, Germany) at 1:10,000 dilutions in blocking buffer for 2 h at room temperature. Subsequent washes included three rinses with 0.1 M PBS‐Tween and a final wash with PBS. Signal visualization was performed using an Odyssey CLx LI‐COR infrared fluorescence imaging system (Biosciences, Germany). Signal intensities were quantified and normalized to β‐actin using LI‐COR Odyssey Image Studio software.

### 2.9. Statistical Analysis

The analyses encompassed five independent experiments, each executed with three technical replicates. The sample size determination for our experiments underwent a meticulous power analysis, leveraging insights from our preliminary studies, prior experiments, and relevant literature sources [72, 73] (all data were included). Statistical analysis was conducted using GraphPad 8.0. Normality was assessed using the Shapiro–Wilk test, and variance was examined when necessary. A two‐tailed unpaired *t*‐test was employed to evaluate the statistical significance between groups (LPS vs. saline). Additionally, a two‐ and three‐way ANOVA analysis was conducted when analyzing FAO data, incorporating LPS treatment, palmitate–BSA stimulation, and etomoxir inhibition of FAO as factors. A Bonferroni post hoc test was applied when a significant interaction occurred between these parameters. Significance was established at *p* < 0.05, and the data were presented as mean ± SEM.

## 3. Results

### 3.1. LPS Toxicity

**FIGURE 1:**
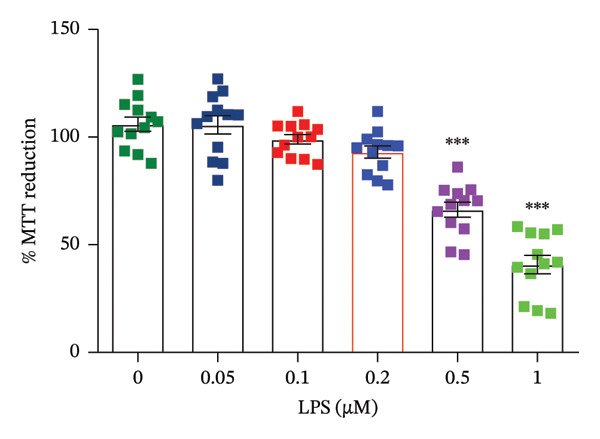
Effect of different doses of LPS (0.005, 0.1, 0.2, 0.5, and 1 μM) on MTT reduction in GT1‐7 hypothalamic cells. Data are expressed as mean ± SEM, ^∗∗∗∗^ *p* < 0.001, Bonferroni post hoc test (*n* = 12 per group).

LPS toxicity was evaluated using the MTT assay after 12 h of incubation at LPS concentrations ranging from 0 to 1 μM. The results showed a significant effect of LPS treatment on the MTT reduction capacity of GT1‐7 cells, an indicator of cell viability (F_(5,66)_ = 56, *p* < 0.0001). Subsequent multiple comparisons revealed that LPS treatment did not significantly affect cell viability at doses below 0.2 μM compared with the vehicle group (*p* > 0.05). However, at doses of 0.5 μM and 1.0 μM, LPS induced a significant decrease in MTT reduction capacity, indicating a loss of cell viability (*p* < 0.05). Based on these findings, the 0.2 μM LPS dose was selected for subsequent assays (Figure 1).

### 3.2. Glycolytic Parameters

The impact of LPS (0.2 μM) on glycolytic function in GT1‐7 cells was assessed by directly measuring the ECAR using an XF analyzer. This instrument enabled the evaluation of key glycolytic flux parameters, including glycolysis, glycolytic capacity, glycolytic reserve, and nonglycolytic acidification (Figure 2(a)). The analysis of these parameters was performed using a two‐tailed unpaired *t*‐test. Our results revealed no significant effect of LPS on glycolysis, glycolytic capacity, and glycolytic reserve compared to the control group (Vehicle) (*p* > 0.05) (Figures 2(b), 2(c), 2(d)). However, LPS did induce a significant increase in nonglycolytic acidification rate compared to the control group (*p* < 0.05) (Figure 2(e)).

FIGURE 2Effect of LPS (0.2 μM) on glycolytic parameters: (a) Representative glycolysis stress test profile showing sequential injections of glucose, oligomycin, and 2‐deoxy‐D‐glucose (2‐DG) and the derived parameters (glycolysis, glycolytic capacity, glycolytic reserve, and nonglycolytic acidification) in Vehicle versus LPS‐treated, (b) glycolysis, (c) glycolytic capacity, (d) glycolytic reserve, (e) nonglycolytic acidification rate in GT1‐7 hypothalamic cell line. Data are expressed as mean ± SEM, ^∗∗^ *p* = 0.0045 < 0.05 in comparison to Vehicle (Veh), *t*‐test (*n* = 5 per group).

**Figure:**
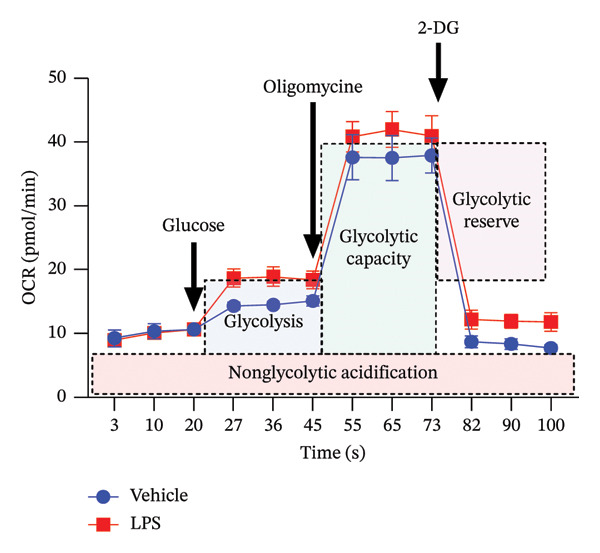
(a)

**Figure:**
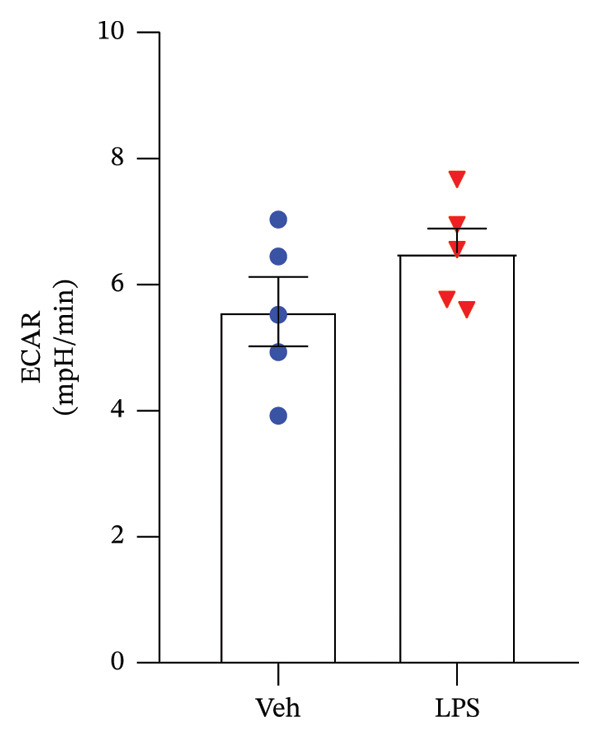
(b)

**Figure:**
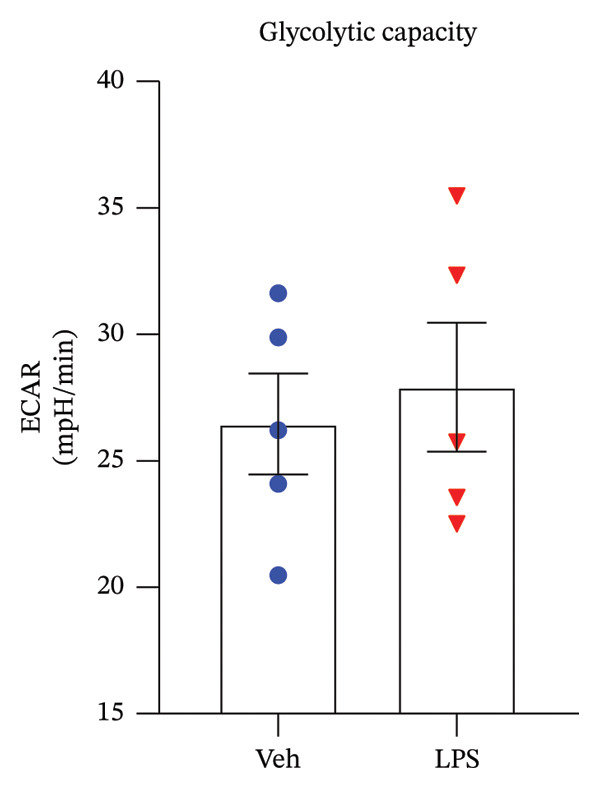
(c)

**Figure:**
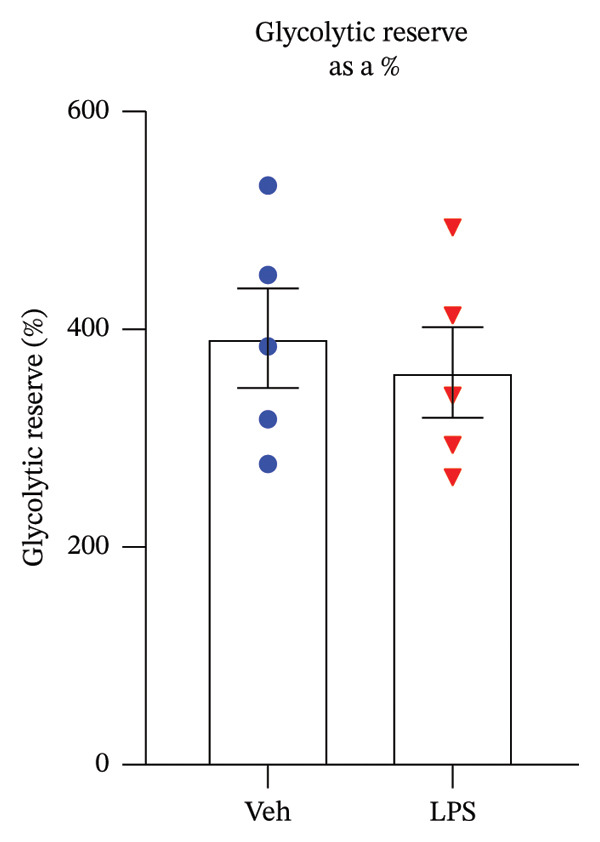
(d)

**Figure:**
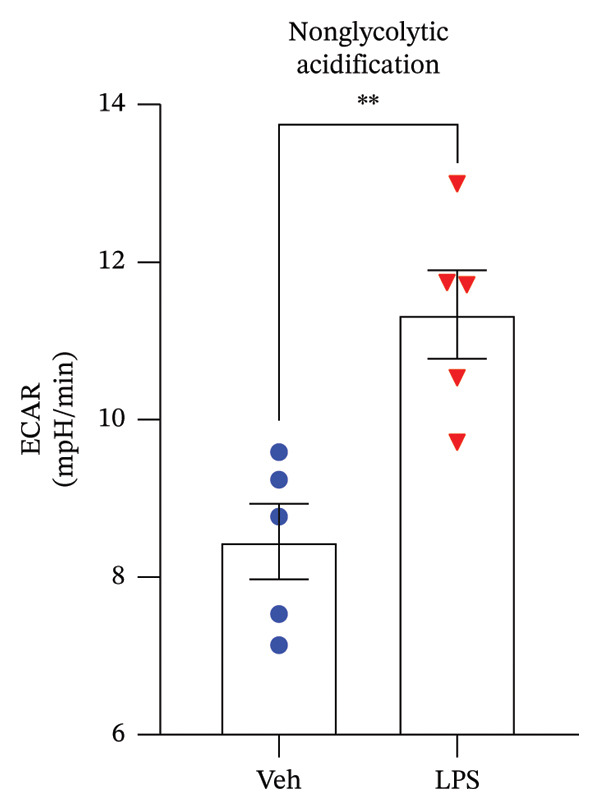
(e)

### 3.3. OXPHOS Parameters

We employed the Seahorse XF Cell Mito Stress test to assess the impact of LPS (0.2 μM) on key mitochondrial function parameters in GT1‐7 cells by directly measuring the OCR (Figure 3(a)). The obtained results revealed a significant increase in nonmitochondrial oxygen consumption (NMOC) and BR activities induced by LPS, accompanied by a reduction in ATP production and spare respiratory capacity (SRC) compared to the control group (*p* < 0.05) (Figures 3(b), 3(c), 3(f), 3(g), respectively). However, it is noteworthy that LPS did not exert any discernible influence on maximal respiration, proton leak, and CE when compared to the control group (*p* > 0.05) (Figures 3(d), 3(e), 3(h), respectively).

FIGURE 3Effect of LPS (0.2 μM) on oxidative phosphorylation: (a) Representative mitochondrial stress test profile showing sequential injections of oligomycin, FCCP, and rotenone/antimycin A and the derived parameters in Vehicle versus LPS‐treated (0.2 μM) GT1‐7 hypothalamic neurons: (b) Nonmitochondrial oxygen consumption. (c) Basal respiration. (d) Maximal respiration. (e) Proton leak. (f) ATP production. (g) Spare respiratory capacity. (h) Coupling efficiency in the GT1‐7 hypothalamic cell line. Data are expressed as mean ± SEM, ^∗^ *p* < 0.05. ^∗∗^ *p* < 0.05. ^∗∗∗^ *p* < 0.001 in comparison to Vehicle (Veh), *t*‐test (*n* = 5 per group).

**Figure:**
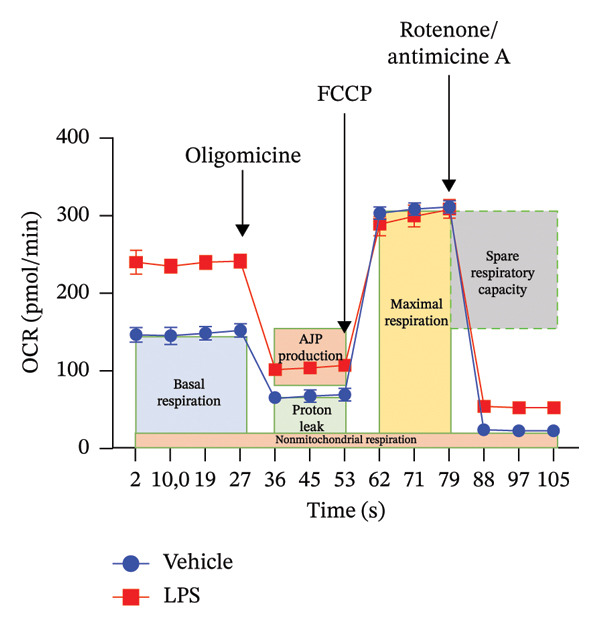
(a)

**Figure:**
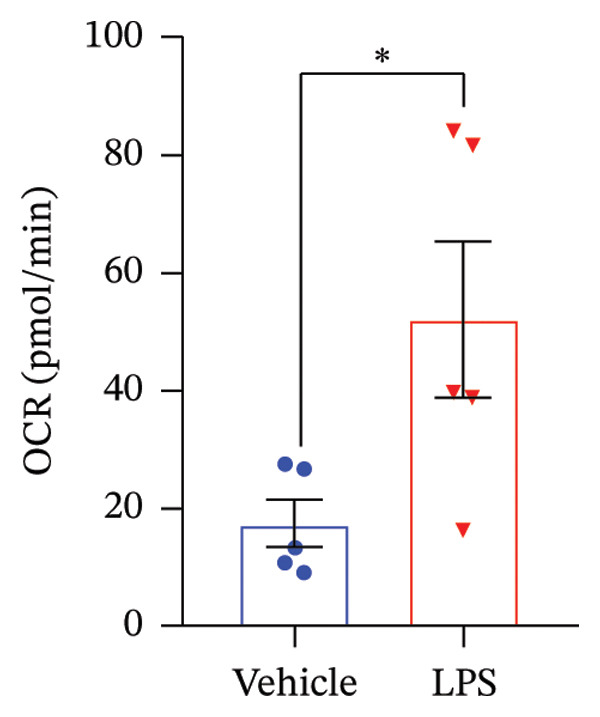
(b)

**Figure:**
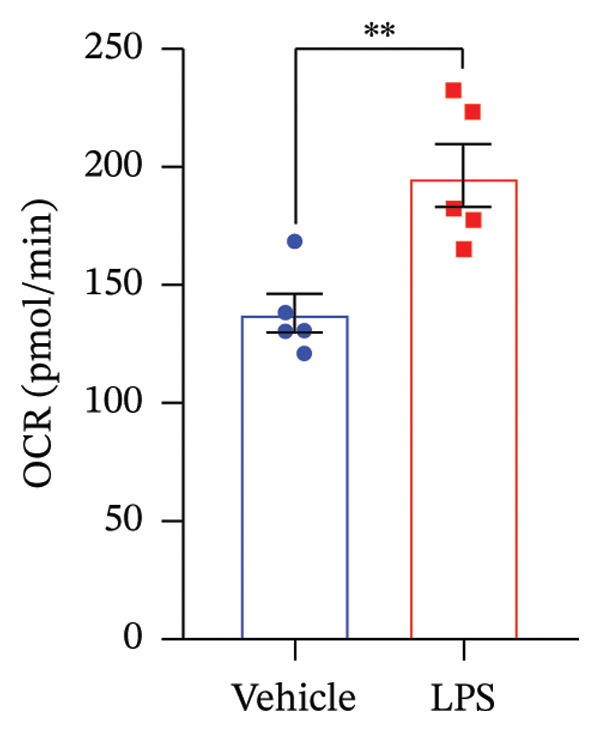
(c)

**Figure:**
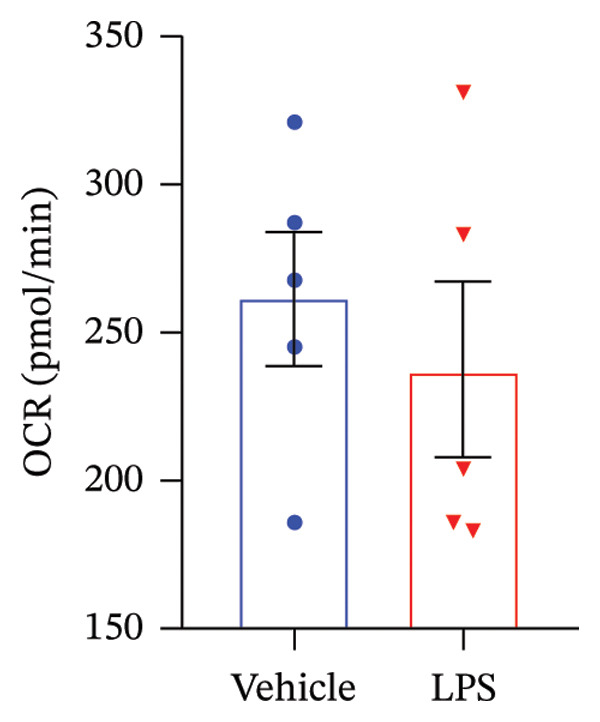
(d)

**Figure:**
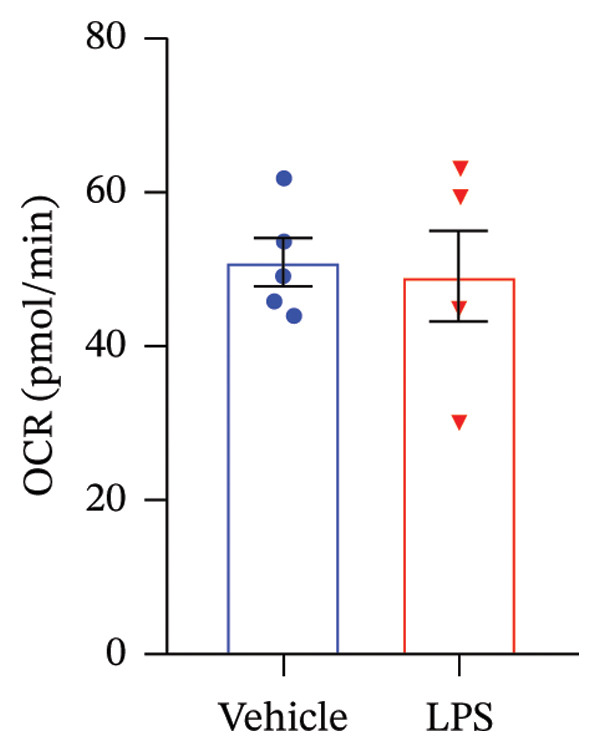
(e)

**Figure:**
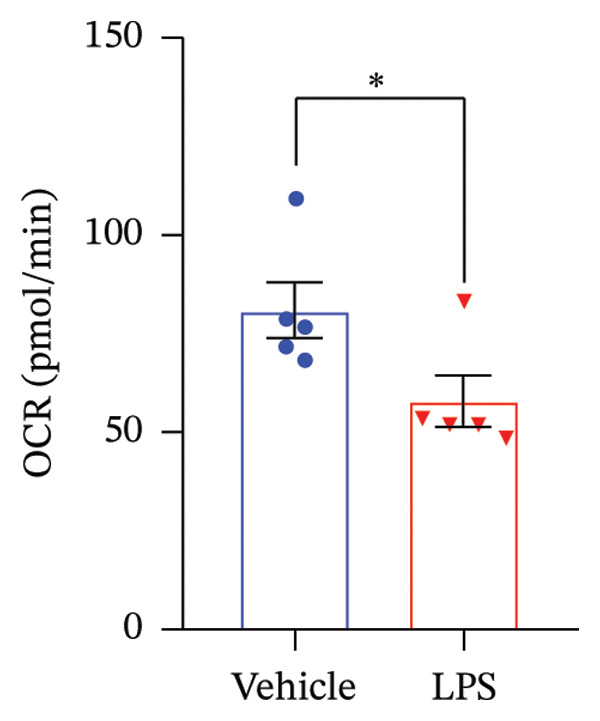
(f)

**Figure:**
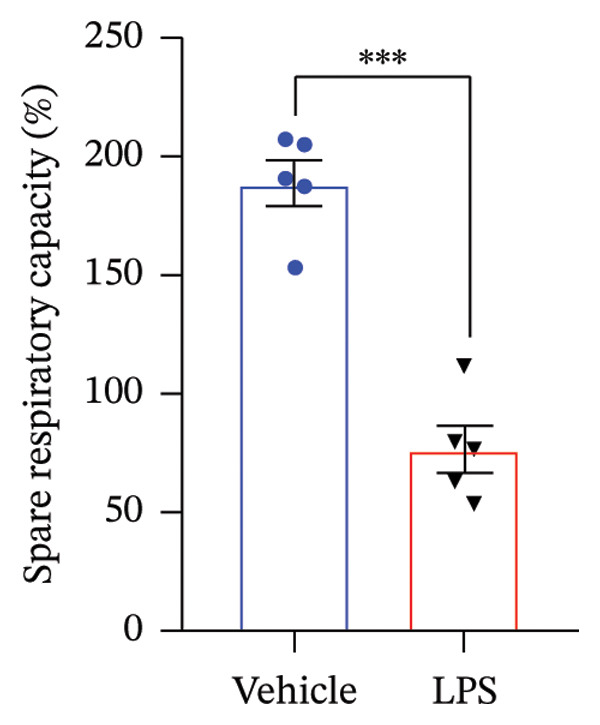
(g)

**Figure:**
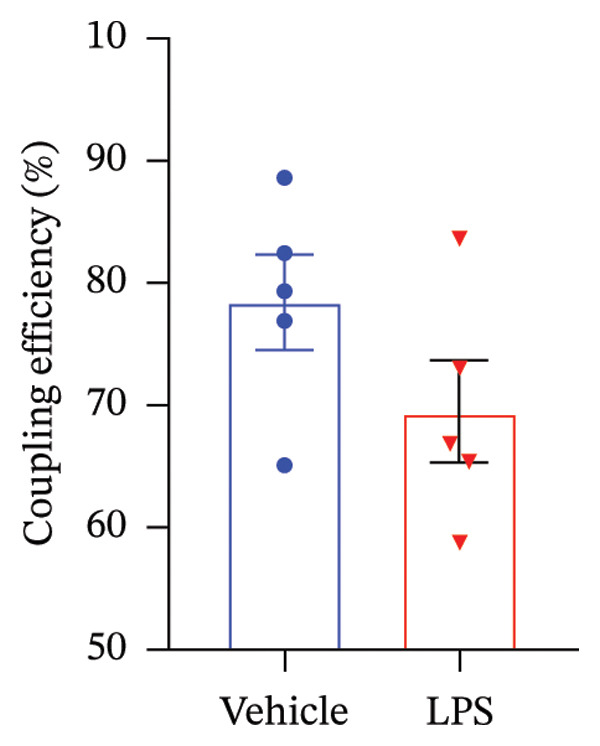
(h)

### 3.4. FAO Parameters

The Seahorse XF Palmitate Oxidative Stress Assay was employed to assess the impact of LPS (0.2 μM) on FAO by examining changes in the OCR within GT1‐7 cells. This assay facilitates the determination of how LPS influences respiratory rates when fatty acids, specifically palmitate, are introduced into the experimental media. It allows for the quantification of the oxidation of both endogenously produced and exogenously supplied long‐chain fatty acids.

The results unveiled noteworthy alterations in FAO dynamics induced by LPS treatment (Figure 4(a)). A two‐way ANOVA analysis revealed a significant effect of LPS treatment on both BR (F_(1,16)_ = 306.6, *p* < 0.0001) and maximal respiration (F(_1,16_) = 6.408, *p* = 0.022). Palmitate–BSA stimulation exhibited a significant effect on maximal respiration (F_(1,16)_ = 27.80, *p* < 0.0001) but not on BR (F_(1,16)_ = 0.791, *p* = 0.378). An interaction between LPS treatment and palmitate–BSA was observed for both BR (F_(1,16)_ = 129.7, *p* < 0.0001) and maximal respiration (F_(1,16)_ = 116.5, *p* < 0.0001). Bonferroni’s post hoc analysis indicated that LPS caused a reduction in both endogenous and exogenous FAO during BR compared to the control group (*p* < 0.05) (Figure 4(b)). However, when mitochondria were uncoupled using FCCP, three‐way ANOVA analysis with etomoxir as a third factor used to inhibit exogenous FAO revealed a significant effect of LPS, etomoxir, and palmitate–BSA (F_(1,32)_ = 166.7, *p* < 0.0001; F_(1,32)_ = 47.10, *p* < 0.0001; F(_1,32_) = 4.907, *p* = 0.034, respectively) on BR, with no significant interaction between these parameters (F_(1,32)_ = 0.160, *p* = 0.6915). However, this three‐way analysis revealed only an effect of palmitate–BSA and etomoxir (F_(1,32)_ = 267.00, *p* < 0.0001; F_(1,32)_ = 32.85, *p* < 0.0001, respectively) with no effect of LPS (F_(1,32)_ = 2.034, *p* = 0.163) and a significant interaction between these parameters (F_(1,32)_ = 15.69, *p* < 0.001) on maximal respiration. Furthermore, analyzing ATP production revealed a significant effect of LPS, etomoxir, and palmitate–BSA (F_(1,32)_ = 112.4, *p* < 0.0001; F_(1,32)_ = 82.21, *p* < 0.0001; F_(1,32)_ = 14.09, *p* < 0.001, respectively) with no significant interaction between these parameters (F_(1,32)_ = 1.328, *p* = 0.257).

FIGURE 4LPS treatment leads to a significant decrease in endogenous fatty acid oxidation and an increase in exogenous fatty acid oxidation, accompanied by increased ATP production, compared to control cells. (a) Kinetic graph of fatty acid oxidation (FAO) in living GT1 neuronal cells using the XF Palmitate Oxidation Stress Test Kit, illustrating the differences in oxygen consumption rate (OCR) associated with endogenous FAO and LPS‐stimulated FAO, measured during basal respiration (b) and maximal respiration (c). Bar charts showcasing the impact of LPS stimulation on GT1‐7 neuronal cells’ basal respiration (d), maximal respiration (e), and ATP production (f). Data presented as mean ± SEM with significance indicated by ^∗^ *p* < 0.05, ^∗∗^ *p* < 0.05 and ^∗∗∗^ *p* < 0.001 in comparison to Vehicle, *t*‐test (*n* = 5 per group).

**Figure:**
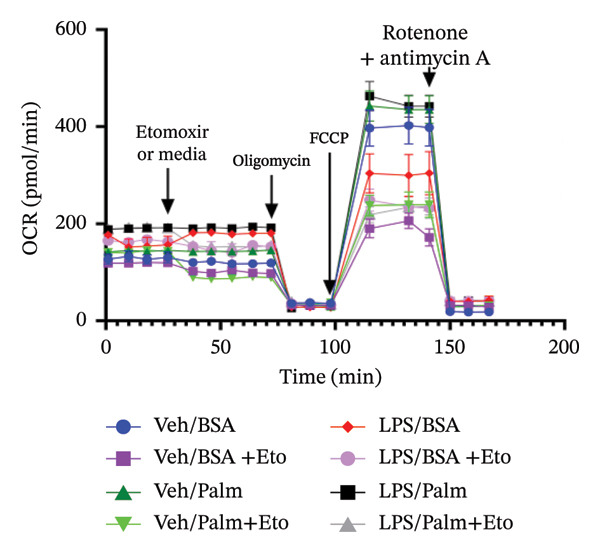
(a)

**Figure:**
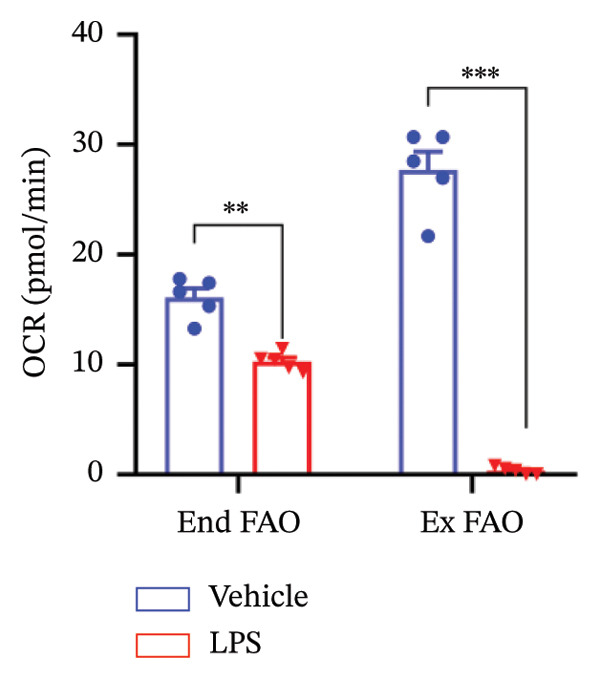
(b)

**Figure:**
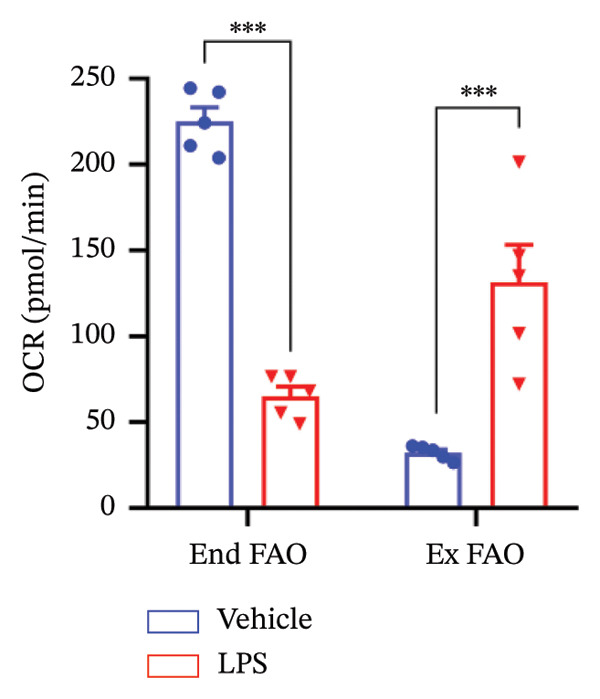
(c)

**Figure:**
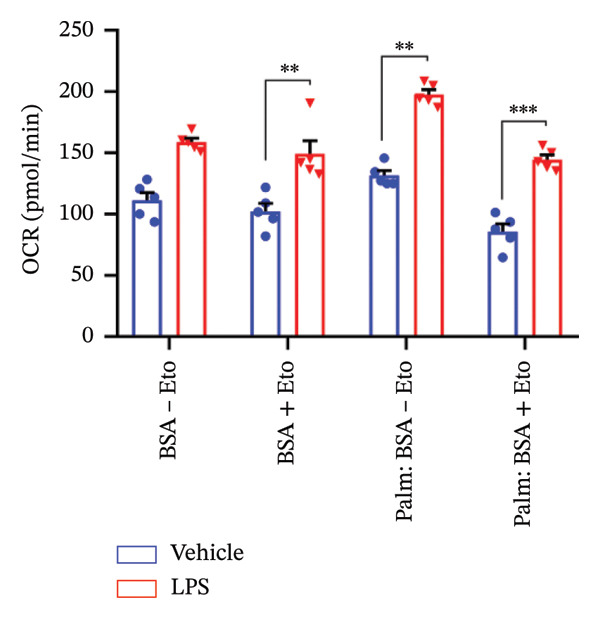
(d)

**Figure:**
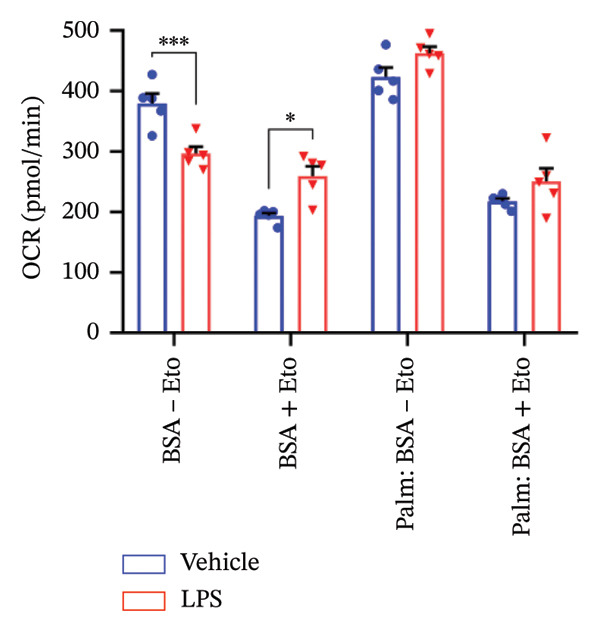
(e)

**Figure:**
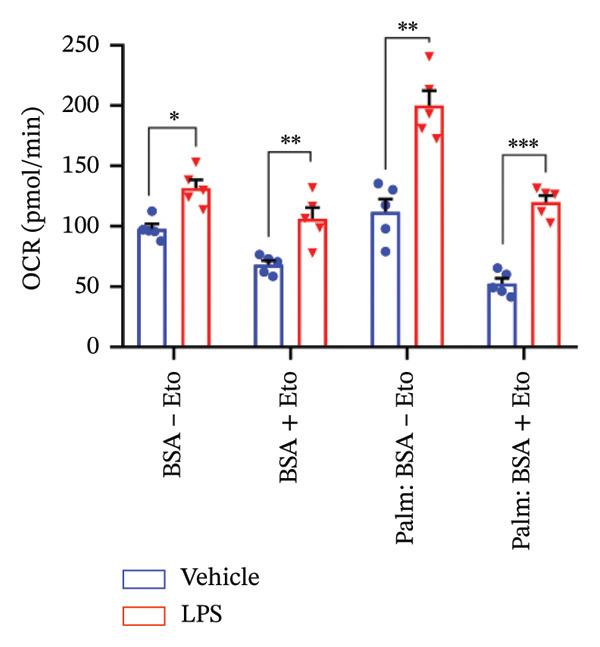
(f)

In this context, results demonstrated a significant decrease in endogenous FAO, coupled with an increase in exogenous FAO after LPS treatment (*p* < 0.05), as shown in Figure 4(c). Notably, when exogenous palmitate was introduced, total mitochondrial oxidation increased in LPS‐treated cells, even in the presence of etomoxir, compared to the control group (*p* < 0.05) (Figure 4(d)). This suggests that LPS may uncouple mitochondria, resulting in elevated O2 consumption not solely attributable to FAO in LPS‐stimulated cells.

Moreover, maximal respiration decreased significantly in the absence of etomoxir, whereas its presence increased in LPS‐treated cells (Figure 4(e)). This effect was observed in the absence of palmitate, with no significant impact (*p* > 0.05), implying that LPS affects not only oxygen consumption related to FAO but also other substrates. Additionally, the heightened OCR due to FAO in LPS‐treated cells correlated with increased ATP production (*p* < 0.05), as illustrated in Figure 4(f).

### 3.5. Impact of LPS on Oxidative Stress, Inflammation, and GnRH Release in GT1‐7 Hypothalamic Cells

Data analysis of our results revealed a striking impact of LPS on various oxidative stress and inflammatory markers and GnRH release by the GT1‐7 hypothalamic cell line. It induced a significant increase in NO and ROS production (Figures 5(a) and 5(b)), indicating heightened oxidative stress. Furthermore, LPS exposure led to a substantial elevation in IL‐6 and TNF levels (Figures 5(c) and 5(d)), indicative of a pronounced inflammatory response. Intriguingly, LPS treatment was also found to reduce GnRH release (Figure 5(e)) with a remarkable significance level of *p* < 0.001. This evidence underscores the complex, multifaceted effects of LPS on the hypothalamic cell line, shedding light on potential mechanisms underlying these molecular changes.

FIGURE 5Assessment of LPS (0.2 µM) impact on oxidative stress, including nitric oxide (NO) (a) and reactive oxygen species (ROS) production (b), along with an exploration of inflammatory markers, specifically interleukin‐6 (IL‐6) (c) and tumor necrosis factor (TNF) (d). Additionally, an investigation into its effects on gonadotropin‐releasing hormone (GnRH) (e) and synaptophysin expression (f) in the GT1‐7 hypothalamic cell line. Data are presented as mean ± SEM with significance indicated by ^∗∗^ *p* < 0.001 compared to Vehicle control and analyzed using a *t*‐test (*n* = 5 per group).

**Figure:**
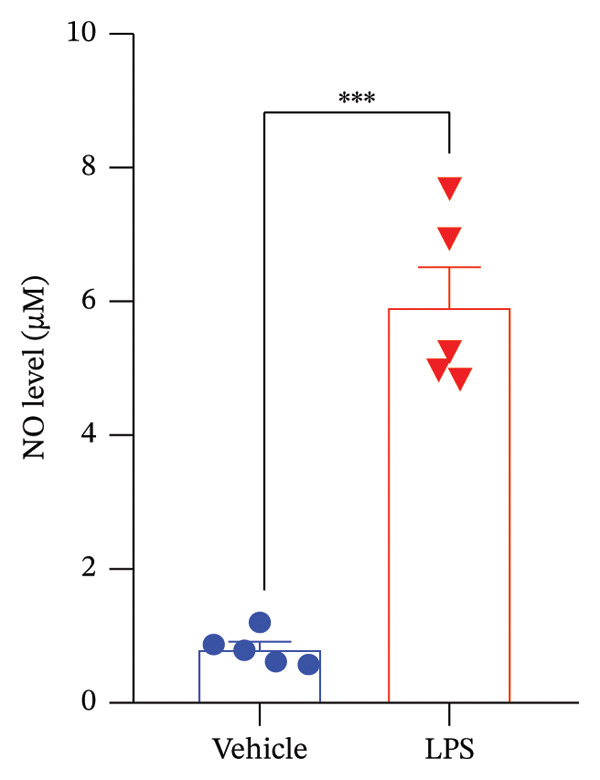
(a)

**Figure:**
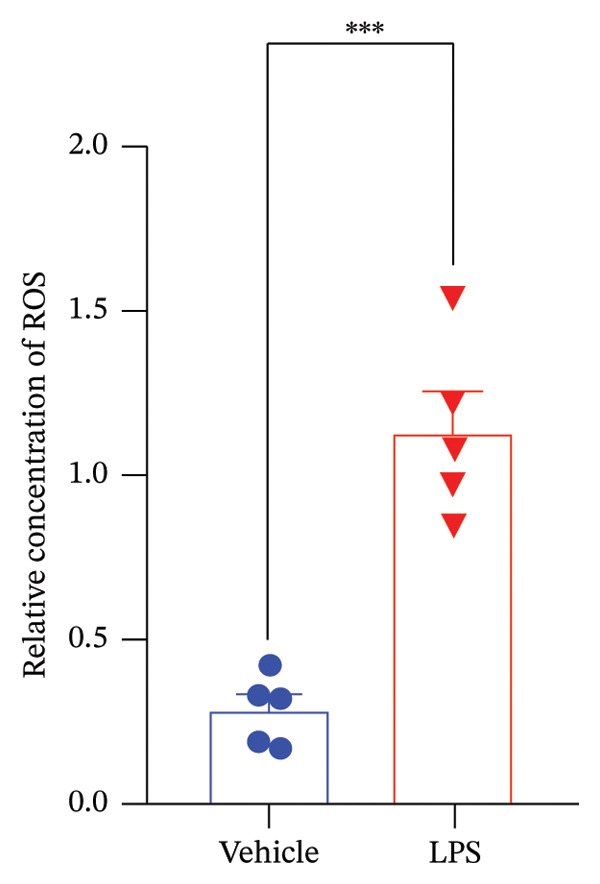
(b)

**Figure:**
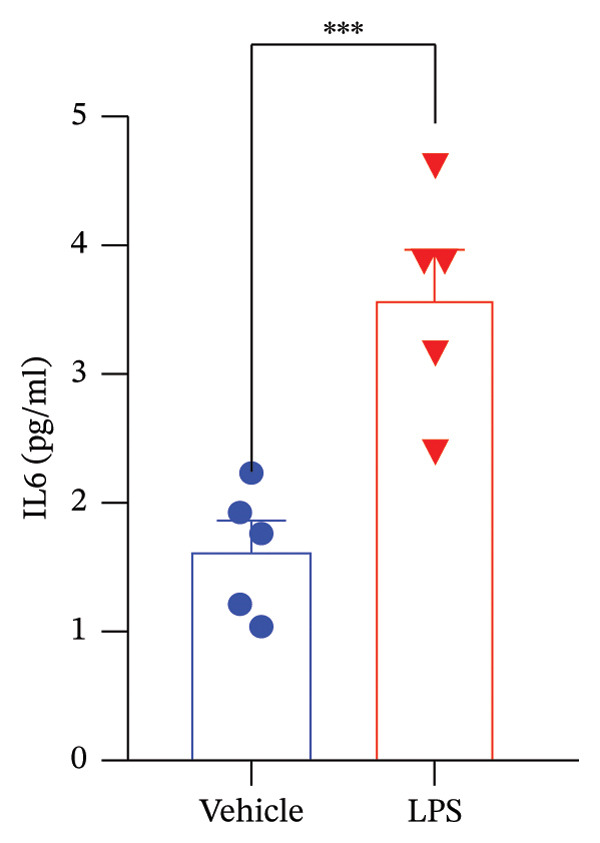
(c)

**Figure:**
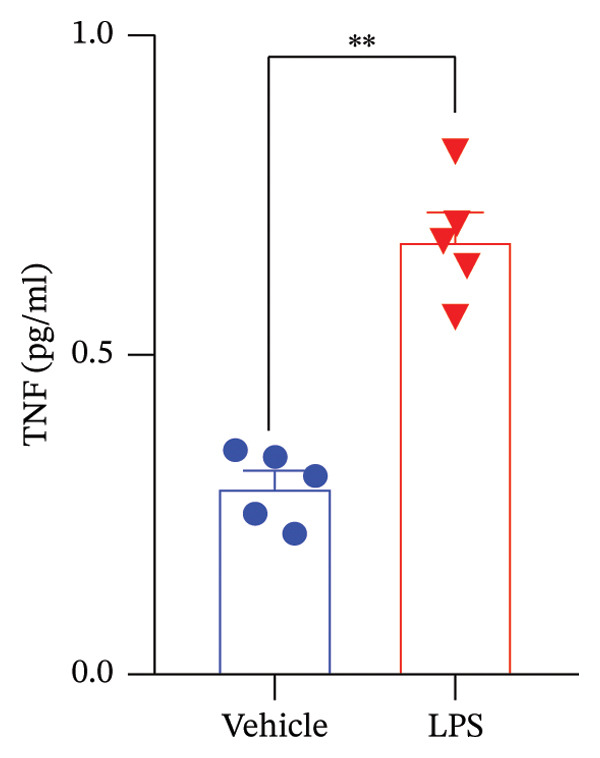
(d)

**Figure:**
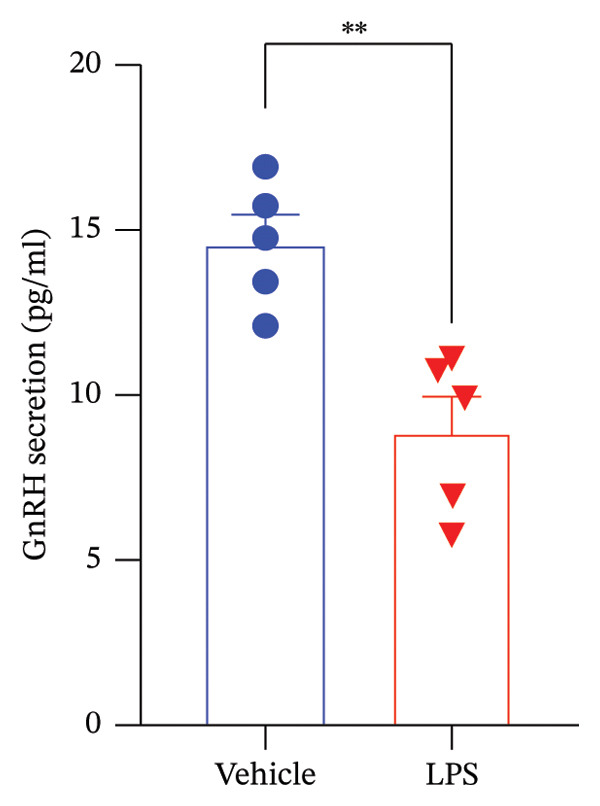
(e)

**Figure:**
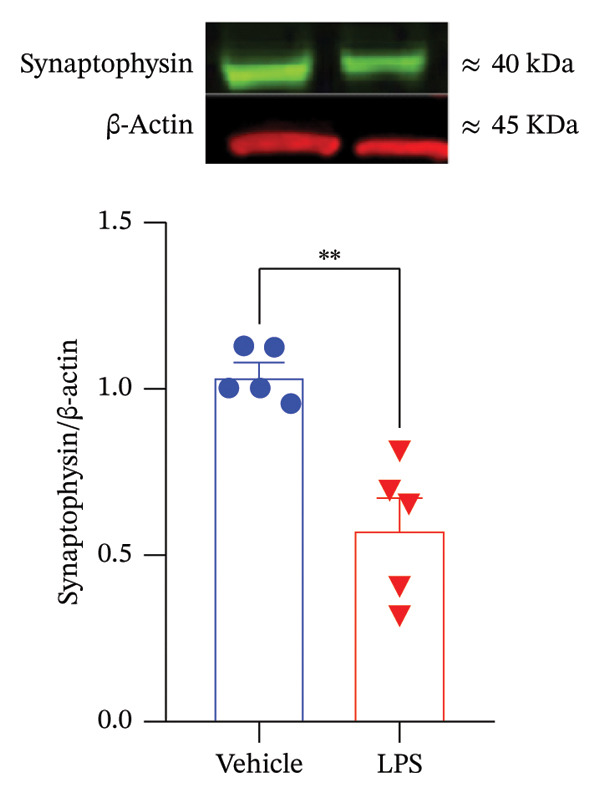
(f)

### 3.6. Impact of LPS on Synaptophysin Expression

Synaptophysin is a biomarker of synaptic plasticity. Data analysis using a two‐tailed unpaired *t*‐test revealed a significant decrease in synaptophysin levels in the LPS‐treated group compared to the control group (*p* < 0.05) (Figure 5(f)). Specifically, the mean ± SEM of synaptophysin signal intensity was 1.042 ± 0.0357 in the vehicle group and 0.579 ± 0.0931 in the LPS‐treated group, confirming the LPS‐induced reduction. Full‐length, uncropped Western blot images are provided in Supporting Figure S1.

## 4. Discussion

The primary endpoint of this study was to evaluate the impact of LPS exposure on a hypothalamic neuronal lineage (GT1‐7 cells), focusing on proinflammatory cytokine secretion and its potential consequences on metabolism, oxidative stress, synaptic plasticity, and functionality. Our findings illuminate the multifaceted effects of LPS on GT1‐7 hypothalamic neurons, providing more insights into the underlying mechanisms.

Our results demonstrate that LPS significantly alters the metabolism of the GT1‐7 cells. Specifically, LPS exposure decreased OXOPHOS and endogenous FAO and increased exogenous FAO and ATP production, with no discernible effect on glycolysis. These metabolic changes occurred concurrently with a substantial elevation in inflammatory markers, including interleukin IL‐6 and TNF‐alpha, as well as oxidative stress biomarkers such as ROS and NO. Additionally, there was a significant reduction in synaptic plasticity, as indicated by decreased synaptophysin levels and functional impairment manifested as a decreased release of GnRH.

In this study, we selected an LPS dose that did not significantly reduce MTT reduction capacity in GT1‐7 cells, indicating preserved viability during the 12‐h exposure. Despite the absence of cytotoxicity, LPS induced a clear inflammatory response, as reflected by increased IL‐6 and TNF‐α levels. These findings add to growing evidence that neurons can mount proinflammatory responses independently of glial cells [74, 75].

It is important to note, however, that MTT primarily detects overt metabolic failure and may not reveal more subtle shifts in mitochondrial function or bioenergetic remodeling occurring at sublethal LPS concentrations. Recent studies show that low‐dose LPS can alter mitochondrial respiration, glycolytic flux, redox balance, and inflammatory gene expression well before any decline in cell survival becomes measurable. For example, Szczesnowski et al. reported enhanced respiration, glycolysis, and IL‐6/SOD2 expression in H9c2 cells exposed to low‐dose LPS [76], while Geng et al. demonstrated broad LPS‐driven metabolic changes in vivo that were not predicted by cell‐death markers alone [77]. These findings support the concept that early metabolic reprogramming can precede overt toxicity. On the other hand, mixed cultures comprising astrocytes, microglia, and neurons have been widely used to study neuroinflammation and neurotoxicity because they more closely reproduce the cellular interactions of the CNS. However, such systems make it difficult to distinguish neuron‐intrinsic responses from those driven by glial‐derived cytokines, ROS, or metabolic substrates [78, 79]. To specifically investigate how neurons respond to inflammatory stress in the absence of glial modulation, we employed a neuronal monoculture model. This reductionist approach enabled us to characterize LPS‐induced changes in neuronal bioenergetics and inflammatory signaling without confounding contributions from glial cells, providing clearer insight into early neuronal‐autonomous metabolic responses.

Within this neuron‐only framework, the observed reduction in OXPHOS without a corresponding increase in glycolysis following LPS treatment suggests early mitochondrial dysfunction or a shift in energy metabolism. Although reduced OXPHOS is classically expected to trigger a compensatory rise in glycolysis (a Warburg‐like response), this pattern is typically described in proliferative or cancer cells [80–82] and does not necessarily apply to neurons. Neurons have limited glycolytic capacity, rely predominantly on mitochondrial ATP production, and, under inflammatory stress, may enter a hypometabolic state or recruit alternative metabolic pathways rather than upregulate glycolysis. The selective decline in OXPHOS observed here may also reflect the absence of astrocyte‐derived lactate, consistent with the astrocyte–neuron lactate shuttle (ANLS) theory, whereby neurons preferentially oxidize lactate supplied by astrocytes [83]. In contrast, our results show that neurons in monoculture efficiently utilize glucose in glycolysis to generate ATP and pyruvate, which enters mitochondria via the MPC complex and is converted into acetyl‐CoA by pyruvate dehydrogenase (PDH) to fuel the TCA cycle and sustain ATP production [84–86].

Furthermore, our results demonstrate that LPS leads to increased utilization of exogenous fatty acids, accompanied by elevated ATP production, while endogenous fatty acid utilization decreases, in a scenario without lactate as an energy fuel. This shift toward utilizing supplemented palmitate for energy generation may be explained by metabolic reprogramming triggered by the high energy demand during LPS‐induced inflammation. The observed increase in exogenous FAO alongside a decrease in endogenous FAO likely reflects an adaptive response; neurons may downregulate the mobilization and use of endogenous fatty acids, such as arachidonic acid, which are precursors for proinflammatory lipid mediators [87, 88]. Instead, increased uptake and oxidation of exogenous fatty acids may help to sustain ATP production when OXPHOS is compromised. Inflammatory signaling can also impair lipolysis and favor upregulation of transporters and enzymes (e.g., CD36, CPT1) that facilitate uptake and catabolism of extracellular fatty acids [88, 89]. This shift may help mitigate excessive eicosanoid‐mediated inflammation and potential cell damage [89, 90]. The decrease in OXPHOS forces neurons to rely on FAO as an alternative pathway. These metabolic adaptations have been described in neuronal and immune cell models subjected to stress or inflammation [88, 89], highlighting a neuron‐specific strategy for balancing energy supply and cellular protection during neuroinflammatory states.

Under inflammatory conditions, endogenous fatty acids, such as arachidonic acid, have roles beyond energy supply, serving as precursors for proinflammatory lipid mediators [91, 92]. Neurons exposed to inflammatory stimuli exhibit enhanced release of arachidonic acid from membrane phospholipids, leading to the production of proinflammatory eicosanoids [93, 94]. The substantial production of ROS observed in our study may result from a combination of factors, including the utilization of fatty acids as an alternative energy source and the absence of efficient antioxidant machinery in neurons. Normally, ROS and NO released by neurons are scavenged by astrocytes, which play a key role in maintaining redox balance [95, 96]. The decline in synaptophysin levels, indicative of synaptic loss, and the reduction in GnRH release suggest a combined action of neuroinflammation and excitotoxicity. This interaction can lead to the release of cytokines, including IL‐6 and TNF‐alpha, from neurons. Furthermore, other inflammatory markers, such as cytosolic phospholipase A2 (cPLA2), secretory PLA2 (sPLA2), and cyclooxygenase‐2 (COX‐2), could be involved in this process [97, 98]. It is worth mentioning that the observed reduction in synaptophysin and GnRH secretion was not accompanied by significant loss of cell viability, suggesting these changes are not mere artifacts of cell damage but instead represent specific functional effects of neuroinflammatory stress. This is further supported by the selective impact on synaptic and neuroendocrine markers, while overall metabolic activity and cell number remained stable. Previous studies have demonstrated that neurons can exhibit decreased synaptophysin and altered GnRH release in response to inflammatory signaling, independent of overt cytotoxicity [78, 79]. Our findings thus indicate a targeted disruption of synaptic plasticity and neuroendocrine function rather than general toxicity.

Even though in vivo studies robustly demonstrate that LPS administration activates microglia as the primary immunocompetent cells of the brain, driving neuroinflammatory responses and subsequent neuronal dysfunction in NDD models [99–101], typically, direct activation of neurons by LPS is considered limited, as microglia and astrocytes mediate the bulk of cytokine production, phagocytic activity, and synaptic remodeling [100, 102]. For example, LPS challenge in mice rapidly activates microglia before any detectable neuronal metabolic or synaptic changes [99, 103]. However, emerging in vitro and cellular neurobiology studies are now revealing that neurons themselves possess TLR4‐dependent inflammatory, oxidative, and metabolic response pathways [104–106]. Our results complement these findings by isolating neuron‐autonomous effects. Our model exhibits distinct metabolic reprogramming and dysfunction in response to LPS exposure, independent of microglial or astrocytic crosstalk. The metabolic and secretory responses found in our GT1‐7 model highlight the importance of neuronal targets and suggest that, while in vivo glial activation dominates the initial response, neuronal mechanisms are both relevant and may present unique therapeutic opportunities, especially for cell‐type‐specific interventions in NDD.

In this study, we selected the GT1‐7 hypothalamic neuron cell line, derived from a murine hypothalamic tumor and widely used in neuroscience research for its advantageous properties. GT1‐7 cells exhibit a neuronal phenotype characterized by the expression of markers such as synaptophysin and GnRH, which indicate synaptic plasticity and neuroendocrine functions. Unlike BDNF, which is primarily associated with neurotrophic support and synaptic remodeling, synaptophysin provides a more direct indication of synaptic integrity and presynaptic function. Furthermore, GT1‐7 cells are hypothalamic neurons with a neuroendocrine profile, and synaptophysin expression is closely linked to their synaptic activity and hormonal release, making it a relevant marker for our study’s objectives. Additionally, these cells can generate neurite‐like extensions that resemble neuronal processes [107]. Their origin from the hypothalamus, a critical brain region regulating various physiological processes and behaviors, further underscores their significance in research [108, 109].

On the other hand, GnRH plays a critical role in regulating the hypothalamic‐hypophysis axis [108]. Mitochondrial dysfunction in GnRH neurons has been linked to impaired GnRH production, potentially disrupting GnRH signaling through this axis [109]. Additionally, hypothalamic stem cells influence the rate of aging [110, 111]. However, it is essential to acknowledge that GT1‐7 cells, being immortalized and tumor‐derived, may exhibit metabolic and inflammatory characteristics that differ from those of primary neurons. Despite these intrinsic limitations, GT1‐7 cells preserve key neuroendocrine, synaptic, and inflammatory properties and have been widely utilized as a practical model to investigate hypothalamic neuronal metabolism and stress signaling. Furthermore, primary hypothalamic neurons pose significant challenges, including limited availability, heterogeneity, short lifespan, and incompatibility with multi‐endpoint metabolic flux assays such as Seahorse analyses used in this study [78, 79, 112].

Despite these considerations, GT1‐7 cells remain a valid model for investigating neuroinflammation, suggesting that a metabolic switch may accompany such inflammation. Literature lacks sufficient data on the metabolic pathways these hypothalamic neurons utilize for energy production under both basal and stress conditions, such as LPS exposure. Consequently, the results obtained may be influenced, and the mechanisms underlying LPS‐induced mitochondrial dysfunction and its effects on neuronal integrity in complex physiological models remain to be elucidated.

Our study is a pioneering effort in the emerging field of “neuro‐immunometabolism,” aimed at clarifying the interplay between neuroinflammation and neuronal metabolism, with a particular focus on neurons as autonomous responders. Our primary objective was to understand how neurons respond to inflammatory conditions on their own, independent of glial cells. Using hypothalamic neuronal lineage (GT1‐7) cells in an LPS‐induced neuroinflammation model, we provide new insights into neuronal‐specific responses to inflammatory stimuli. This raises the hypothesis that neuronal responses to inflammation may precede or occur independently of glial activation, highlighting the need for further neuron‐focused studies.

It is noteworthy that while our results suggest a link between inflammation and metabolic changes, we acknowledge that they are not sufficient to establish a direct causal relationship. This link can be strengthened in future studies by experimentally manipulating key metabolic pathways using pharmacological modulators of mitochondrial function, glycolysis, or FAO and assessing whether restoring metabolic balance rescues synaptophysin or GnRH output. Moreover, Time‐course analyses and genetic tools (e.g., siRNA/CRISPR targeting metabolic enzymes) would help determine whether metabolic changes precede synaptic impairment. Combining these approaches with quantitative synaptic assays (synaptophysin/PSD‐95 immunoblotting, imaging, and electrophysiology) and complementary in vivo neuroinflammation models would provide mechanistic clarity and help determine whether preventing metabolic dysfunction can mitigate synaptic loss. Also, we recommend a comprehensive examination of the decrease in OXPHOS and its contradictory association with increased levels of ROS and NO, as our findings suggest an inefficient antioxidant machinery in neurons, which could contribute to their vulnerability under inflammatory conditions.

In conclusion, our results underscore the need for further research to elucidate the complex pathways by which LPS affects neuronal metabolism, function, and survival. Addressing these limitations in future studies will be crucial for advancing our understanding of NDDs and identifying potential therapeutic targets.

## Author Contributions

Mohsine‐Ali El‐Hamri wrote the first draft of the manuscript and reassembled the experimental data.

Mohsine‐Ali El‐Hamri and Rafik El‐Mernissi conducted the cell culture experiments, maintained the cell lines, and performed cell metabolic profiling.

Meriem Lahmouad revised and edited the manuscript for intellectual content. Khayelihle Brian Makhathini and Oualid Abboussi conducted the statistical analyses and interpreted the results.

Hanane Khalki and Jihane Zerrouk contributed to data validation and assisted in the critical revision of the manuscript.

Lhoussain Hajji provided general supervision throughout the research project.

Oualid Abboussi and Khayelihle Brian Makhathini supervised the research and ensured all experimental protocols were followed accurately.

## Funding

The experiments for this study were conducted using resources provided by Mohammed V University, specifically within the Department of Physiology and Physiopathology Team, Genomic of Human Pathologies Research. No external funding was received or utilized for this research; the work was enabled by institutional support and access to existing lab equipment.

## Disclosure

All authors reviewed, revised, and approved the final version of the manuscript.

## Ethics Statement

We confirm that institutional approval for this study was obtained from the head manager of the laboratory within the Department of Physiology and Physiopathology Team, Genomic of Human Pathologies Research. Ethical approval was not applicable for this study, as it did not involve human/animal subjects or sensitive materials. All procedures were conducted in accordance with the institutional guidelines.

## Conflicts of Interest

The authors declare no conflicts of interest.

## Supporting Information

Supporting Figure S1. Uncropped Western blot images corresponding to synaptophysin analysis. Full‐length and unprocessed electrophoretic images of the Western blot used for the analysis of synaptophysin expression in GT1‐7 hypothalamic neurons treated with Vehicle (Veh) or LPS. The left panel shows the immunoblot for synaptophysin, while the right panel shows the corresponding total protein/loading control (∼45 kDa) used for normalization. Lane identities correspond to those presented in the main figure (Veh and LPS conditions). These uncropped blots are provided to ensure transparency and to allow visualization of the entire membrane and all detected bands. Note: No lanes were removed, and no image processing was applied other than uniform brightness and contrast adjustment to the entire image.

## Supporting information


**Supporting Information** Additional supporting information can be found online in the Supporting Information section.

## Data Availability

The manuscript comprehensively encompasses all available data and materials, which are also accessible upon request from the corresponding author.
