# Supplementary material for: Roles of Oxidative Phosphorylation and Fatty Acid Oxidation in Neuroinflammation Induced by Lipopolysaccharide in Hypothalamic Neuronal Cells
Source: Int J Inflam. 2026 Apr 15;2026:6298730. doi: 10.1155/ijin/6298730 (PMC13081690; doi:10.1155/ijin/6298730)
Supplement: Supplementary file 1 — Supporting Information Additional supporting information can be found online in the Supporting Information section. [file IJIN-2026-6298730-s001.pdf]

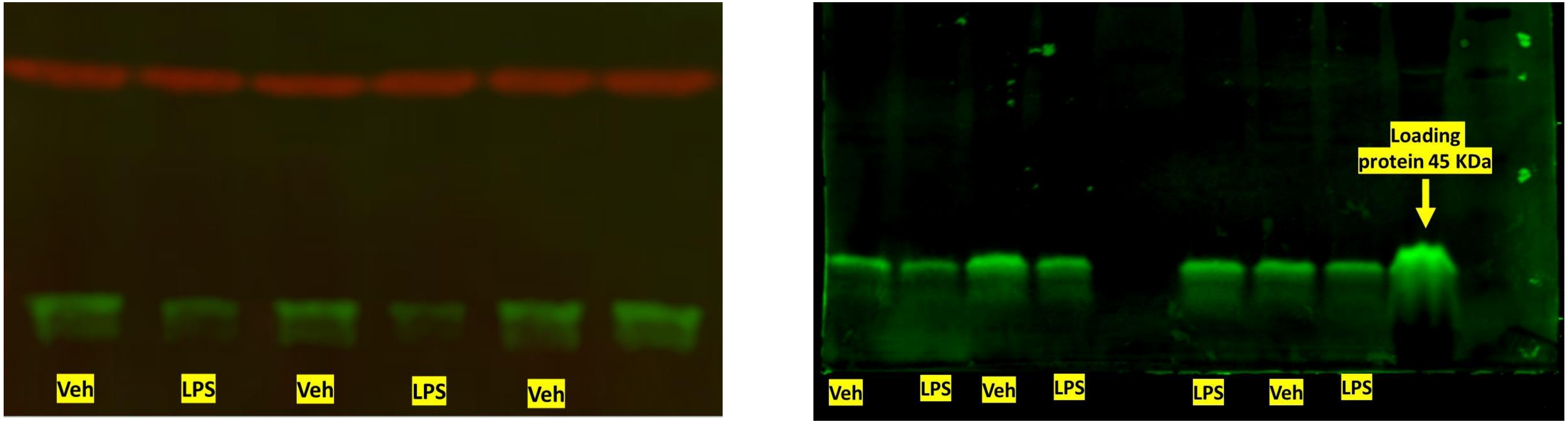

Supplementary Figure S1. Uncropped Western blot images corresponding to synaptophysin analysis. *Full-length and unprocessed electrophoretic images of the Western blot used for the analysis of synaptophysin expression in GT1-7 hypothalamic neurons treated with vehicle (Veh) or lipopolysaccharide (LPS). The left panel shows the immunoblot for synaptophysin, while the right panel shows the corresponding total protein/loading control (~45 kDa) used for normalization. Lane identities correspond to those presented in the main figure (Veh and LPS conditions). These uncropped blots are provided to ensure transparency and to allow visualization of the entire membrane and all detected bands.*

Note: *No lanes were removed, and no image processing was applied other than uniform brightness and contrast adjustment to the entire image.*
